# Optimizing Gō-MARTINI Coarse-Grained Model for F-BAR Protein on Lipid Membrane

**DOI:** 10.3389/fmolb.2021.619381

**Published:** 2021-02-22

**Authors:** Md. Iqbal Mahmood, Adolfo B. Poma, Kei-ichi Okazaki

**Affiliations:** ^1^ Department of Theoretical and Computational Molecular Science, Institute for Molecular Science, National Institutes of Natural Sciences, Okazaki, Japan; ^2^ Institute of Fundamental Technological Research, Polish Academy of Sciences, Warsaw, Poland

**Keywords:** molecular dynamics simulation, MARTINI force field, Gō model, membrane remodeling, Pacsin

## Abstract

Coarse-grained (CG) molecular dynamics (MD) simulations allow us to access much larger length and time scales than atomistic MD simulations, providing an attractive alternative to the conventional simulations. Based on the well-known MARTINI CG force field, the recently developed Gō-MARTINI model for proteins describes large-amplitude structural dynamics, which has not been possible with the commonly used elastic network model. Using the Gō-MARTINI model, we conduct MD simulations of the F-BAR Pacsin1 protein on lipid membrane. We observe that structural changes of the non-globular protein are largely dependent on the definition of the native contacts in the Gō model. To address this issue, we introduced a simple cutoff scheme and tuned the cutoff distance of the native contacts and the interaction strength of the Lennard-Jones potentials in the Gō-MARTINI model. With the optimized Gō-MARTINI model, we show that it reproduces structural fluctuations of the Pacsin1 dimer from atomistic simulations. We also show that two Pacsin1 dimers properly assemble through lateral interaction on the lipid membrane. Our work presents a first step towards describing membrane remodeling processes in the Gō-MARTINI CG framework by simulating a crucial step of protein assembly on the membrane.

## Introduction

Large-scale shape changes of membrane structures in the cell are important in many biological processes such as endocytosis, exocytosis and vesicle trafficking (McMahon and Gallop, 2005). These membrane remodeling processes emerge from the interplay between lipids and proteins (McMahon and Gallop, 2005; Suetsugu et al., 2014; Bassereau et al., 2018). Because of dynamic nature of these processes, molecular dynamics needs to be clarified to understand their mechanisms. The molecular dynamics (MD) simulation is a powerful tool to study the dynamic processes at molecular level (Marrink et al., 2019). However, the conventional all-atom (AA) MD has limitations in size and time scales. It is too costly to simulate a large system of membrane remodeling that contains large lipid membrane, large number of proteins and solvent molecules with a time scale longer than microseconds by AA MD. Thus, the coarse-grained (CG) model that represents a group of atoms by a single bead, offers a good alternative to study large membrane remodeling processes (Marrink et al., 2019).

Various CG models of lipids and proteins have been developed previously (Tozzini, 2005; Ayton et al., 2007; Klein and Shinoda, 2008; Takada, 2012; Marrink et al., 2019). For lipids, there are reasonably accurate and transferable CG models such as MARTINI and SPICA (Marrink and Tieleman, 2013; Marrink et al., 2019; Seo and Shinoda, 2019). For proteins, there are structure-based models such as elastic network (EN) and Gō models (Tozzini, 2005; Takada et al., 2015). However, relatively less effort has been made on CG models of the combined protein-membrane system, which should be important for describing the membrane remodeling processes. For example, the popular MARTINI model introduces the EN model to proteins (denoted as EN-MARTINI) (Periole et al., 2009), which assumes unbreakable harmonic bonds, and thus, is unable to describe large-scale motions such as protein unfolding or conformational changes between two stable conformations. These large-scale motions should be important to describe realistic dynamics of the membrane remodeling. The recently developed Gō-MARTINI addressed this issue by replacing the harmonic potential with the Lennard-Jones (LJ) potential based on the contact map of the native protein structure (Poma et al., 2017). The Gō-MARTINI model combines the flexibility of the Cα-based Gō-like model for the sampling of large conformational changes in proteins (Okazaki et al., 2006, 2012; Okazaki and Takada, 2008; Poma et al., 2018, 2019; Senapati et al., 2019) and the versatility of the MARTINI force field that allows the description of different biomolecules, (e.g. lipids, polysaccharides, polymers and nucleic acids) at almost atomistic resolution (Marrink and Tieleman, 2013; Uusitalo et al., 2015; Souza et al., 2020). At the moment, some studies including the original developmental work have used Gō-MARTINI for protein-only systems (Poma et al., 2017; Souza et al., 2019), and not much has been done for protein-membrane systems. Only a few studies have used Gō-MARTINI for protein-membrane systems (Thallmair et al., 2019).

In this study, we apply the Gō-MARTINI model to the F-Bin/Amphiphysin/Rvs (F-BAR) protein Pacsin1 as a model protein that is involved in the membrane remodeling. Pacsin proteins are involved in clathrin-mediated endocytosis, actin polymerization and neuronal development. In the previous study, we showed that Pacsin1 induces and senses the membrane curvature in the EN-MARTINI framework (Mahmood et al., 2019). However, it was found that structural fluctuations of Pacsin1 in the EN-MARTINI model are underestimated, which can affect the stability of the protein complex (Baaden and Marrink, 2013; Stark et al., 2013). Since the association and dissociation of protein complexes play a crucial role in membrane remodeling processes, the underestimated fluctuations can lead to an incorrect description of the processes. Here, in order to overcome the limitations of the EN model, we introduced a simple cutoff scheme of the Gō-MARTINI and tuned the parameters to reproduce structural fluctuations of Pacsin1 on the lipid membrane observed in the AA simulations. We further show that Pacsin1 properly assembles on the membrane with the optimized parameters. This study is a first step toward describing realistic dynamics of the membrane remodeling in the Gō-MARTINI framework.

## Materials and Methods

### All-atom MD Simulations

For our study, we have chosen the human Pacsin1 F-BAR domain crystal structure with the PDB ID 3HAH (Wang et al., 2009). The structure consists of two monomers with some missing residues. MODELLER (Martí-Renom et al., 2000; Webb and Sali, 2016) was employed for modeling the Pacsin1 dimer missing residues (first monomer: T172-L191, second monomer: T172-K194) without referring to a homologous structure. The missing residues at the N- and C-terminal parts were not considered in the simulations. The N-terminal part consists of 15 residues with four negatively charged amino acids. Although the role of the N-terminal part remains unclear, it is unlikely that this highly negatively charged region is involved in interaction with the negatively charged lipid head groups of the membrane. The C-terminal part consists of the central linker and SH3 domain, which have been experimentally shown to decrease the membrane transformation activity (Wang et al., 2009). First, the coordinates of mixed lipid bilayer (POPC 20%, POPE 20%, POPS 60%) (Wang et al., 2009) were generated by the membrane builder tool of CHARMM-GUI (Sunhwan et al., 2008; Wu et al., 2014). Then, Pacsin1 structure was placed on the lipid bilayer using VMD (Humphrey et al., 1996). TIP3P water molecules and neutralizing ions of 0.15 M Na^+^ and Cl^−^ were added to the system, making a periodic boundary box (x:23 nm, y:23 nm and z:18 nm) with the total number of atoms 909109. The CHARMM36 force field was used for lipid bilayers and protein (Venable et al., 2010). The simulation procedure was the same as that of our previous work (Mahmood et al., 2019). The 500 ns production runs were conducted at a temperature of 310 K and a pressure of 1 atm.

### Conventional EN-MARTINI Simulations

The MARTINI coarse-grained (CG) molecular dynamics (MD) simulations described in this paper were performed with the GROMACS-2018 simulation package (Abraham et al., 2015) (www.gromacs.org). The CG model of the Pacsin1-membrane system was constructed using the MARTINI force field version 2.2 (Marrink et al., 2007; Monticelli et al., 2008; De Jong et al., 2013; Marrink and Tieleman, 2013) with additional EN potential for the protein. The EN model was used to maintain the secondary and tertiary structures of proteins based on definition by the DSSP algorithm (version 2.2.1) (Kabsch and Sander, 1983). The spring constant of 500 kJ mol^−1^nm^−2^, the lower and upper elastic bond cut-off to 0.5 and 1.2 nm, respectively (Periole et al., 2009; Mahmood et al., 2019) were applied to the Pacsin1 crystal structure (PDB ID 3HAH) (Wang et al., 2009). The numbers of the elastic bonds from this definition were 2688 for chain A and 2646 for chain B. The numbers are different between the two chains, reflecting a slight difference in their structures. A possible approach to improve the definition of the elastic bonds, as well as the Gō native contacts, is mentioned in DISCUSSION. The protein CG structure and topology were generated using the script “martinize.py” (De Jong et al., 2013). Then, we used a script “insane.py” (Wassenaar et al., 2015) for constructing the flat lipid membrane, aligning proteins on the membrane, generating water and ions. The lipid membrane consists of mixed lipids POPC, POPE and POPS (20%:20%:60%). The systems were hydrated using CG water beads and made charge neutral by addition of an appropriate number of ions with 0.15 M Na^+^ and Cl^−^. The total number of beads in the system was about 292743 CG beads and the system box size was x:60 nm, y:30 nm and z:20 nm. Energy minimization of the system was performed with 5,000 steps of the steepest descent method. After minimization, the system was equilibrated for 0.5 ns in the NPT ensemble using the Berendsen pressure coupling (Berendsen et al., 1984). The following production simulations were run at 300 K with separate temperature coupling for the solvent, lipids and protein using the stochastic rescaling scheme (Bussi et al., 2007) (τ = 1 ps) and the Parrinello-Rahman (Parrinello and Rahman, 1981) semiisotropic pressure coupling at 1 bar. A time step of *dt* = 20 fs was used. The reaction field electrostatics and LJ potentials were shifted to zero at the cut-off distance of 1.2 nm.

### Gō-MARTINI Simulations

**FIGURE 1:**
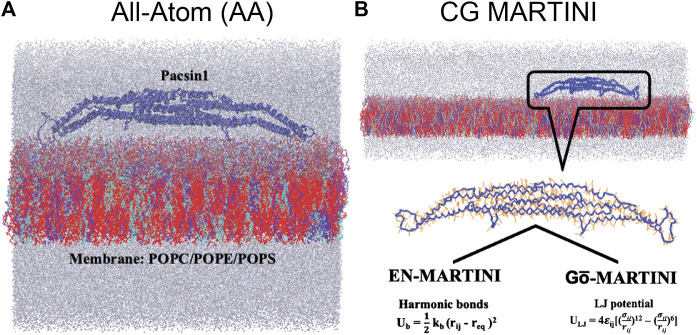
All-atom (AA), coarse-grained (CG) EN-MARTINI and Gō-MARTINI molecular dynamics systems of the protein and membrane are shown **(A)** and **(B)** respectively. Blue color carton represents Pacsin1 protein. Three kind of lipids composition (cyan: 20% POPC, blue: 20% POPE and red: 60% POPS). Water molecules and ions are represented in gray color.

In the Gō-MARTINI simulations, we have replaced the harmonic bonds of the commonly used EN model with the LJ potential based on the contact map of the native protein structure as in Gō models (Poma et al., 2017). There are several types of contact maps with different definitions of native contacts (Clementi et al., 2000; Koga and Takada, 2001; Sułkowska and Cieplak, 2008; Noel et al., 2012). The original Gō-MARTINI adopts the atomic overlap criterion (OV) and chemistry-based rCSU for definition of the native contacts (Sułkowska and Cieplak, 2008; Wołek et al., 2015; Poma et al., 2017). With this definition, the Pacsin1 conformation became distorted during the simulations with respect to the conformations observed in the all-atom simulations (Supplementary Figure S1). Although it worked for globular proteins (Poma et al., 2017), the OV + rCSU definition of the native contacts might result in an unnatural conformation for extended structures like Pacsin1 (Figure 1A) in a balance between the native contacts and the non-native interactions of the MARTINI force field. Thus, we adopt a simpler cutoff scheme for the native contact definition as described in the following. First, all *i* th and (*i* + 3) th amino-acid pairs in the sequence are considered as the native contact, providing a similar interaction as the dihedral term in the typical Gō models. Then, for *i* th and *j*>*i* + 3 th amino acid pairs, if the residue-residue minimum distance considering all non-hydrogen atoms is below a cutoff distance, the pair is considered as the native contact. The cutoff distance of 4.5 Å, 5.0 Å and 5.5 Å were tested. As shown in Supplementary Table S1, the number of the native contacts significantly increased with the new definition, while keeping almost all contacts from the OV + rCSU definition. The number of the native contacts, however, are less than the number of the elastic bonds used in the EN-MARTINI (see the previous section). The numbers of the native contacts are different between the two chains, reflecting a slight difference in their crystal structures. For the cutoff distance of 5.0 Å, the number of the common contacts shared between the two chains is 793, which is 93% and 94% of the total contacts of the chain A and chain B, respectively. The rest of the contacts is unique to each chain. Note that the native contacts were defined for each chain of Pacsin1 and no native contact was defined between the two chains. Thus, it would be interesting to see if the interface structure is maintained only with the MARTINI force field. To check the interface structure between the two chains, we calculated the fraction of the “virtual” (that is, not considered in the model potential) native contacts at the interface present during the Gō-MARTINI simulations (Q_AB_) (Poma et al., 2017). The virtual native contacts were defined in the same way as the intra-chain contacts with the cutoff distance 5.0 Å. The native contact between residues i,j is considered to be present when its distance satisfies $r_{ij}<1.5\sigma_{ij}\approx 1.34r_{ij}^{0}$ (see below for definitions of $\sigma_{ij}$ and $r_{ij}^{0}$). The backbone beads (BB), that is, Cα positions, were used for the interaction sites. In the LJ potential, the parameter *ε*_*ij*_ controls the strength of the native contact interaction in unit of *ε*, where *ε* = 6.276 kJ mol^-1^. This value corresponds to the typical energy scale of hydrogen bonds in proteins (Poma et al., 2015) and λ in the native contact energy, ε_*ij*_ = λ ε, is a tunable parameter. In this study, λ = 1.0, 1.5 were tested. The LJ potential of the native contacts is defined as,

$$U_{LJ}=\sum_{i,\;j\;\in \text{Native contacts}}4\varepsilon_{ij}\left[{\left(\frac{\sigma_{ij}}{r_{ij}}\right)}^{12}-{\left(\frac{\sigma_{ij}}{r_{ij}}\right)}^{6}\right],$$ (1)

where $\sigma_{ij}=r_{ij}^{0}/2^{1/6}$ with $r_{ij}^{0}$ being the Cα-Cα distance of the native-contact pair in the native structure. The bond angle term is another factor in Gō models, biasing towards the native structure. The bond angle force constants for helices and the other secondary structures were set to K_BBB_ = 700 kJ mol^-1^ and K_BBB_ = 20 kJ mol^-1^, respectively (Monticelli et al., 2008). For proline residues in the helix kink region (Pro 145 and Pro221), the force constants were set to K_BBB_ = 20 kJ mol^-1^. Our modified version of “go_martinize.py” script was used to generate the protein coarse-grained structure and topology. The script is available on GitHub (https://github.com/OkazakiLab/Go-MARTINI). The following system setup and simulations were done in the same way as the conventional EN-MARTINI described in the previous section.

### Principal Component Analysis

The principal component analysis (PCA) was performed to identify large-amplitude conformational changes of Pacsin1 from simulation trajectories. We only considered Cα positions of the AA or backbone-bead (BB) positions of the MARTINI simulations, after Pacsin1 structure was superimposed in the trajectories. Then, a covariance matrix was calculated and diagonalized to obtain eigenvalues and eigenvectors in the order of their contributions to the conformational changes. First, PCA was performed for each simulation: AA, EN-MARTINI, and Gō-MARTINI. In order to compare the PCA results, we calculated the root mean square inner product (RMSIP) (Amadei et al., 1999),

$$\text{RMSIP}=\sqrt{\frac{1}{10}\sum_{i=1}^{10}\sum_{j=1}^{10}{\left(u_{i}\cdot v_{j}\right)}^{2}},$$ (2)

where $u_{i}$ and $v_{j}$ represent eigenvectors obtained by two different PCAs, and the first 10 eigenvectors were considered. The RMSIP quantifies how much two simulation trajectories are overlapped in a subspace described by the first 10 eigenvectors. Another way to quantify an overlap among multiple trajectories is to perform a single PCA using all trajectories and project them onto common principal components (Martín-García et al., 2015). We performed the PCA using all three simulations, after superimposing Pacsin1 structure in all three trajectories.

## Results

### Structural Flexibility of Pacsin1 With EN-MARTINI and Gō-MARTINI

**FIGURE 2:**
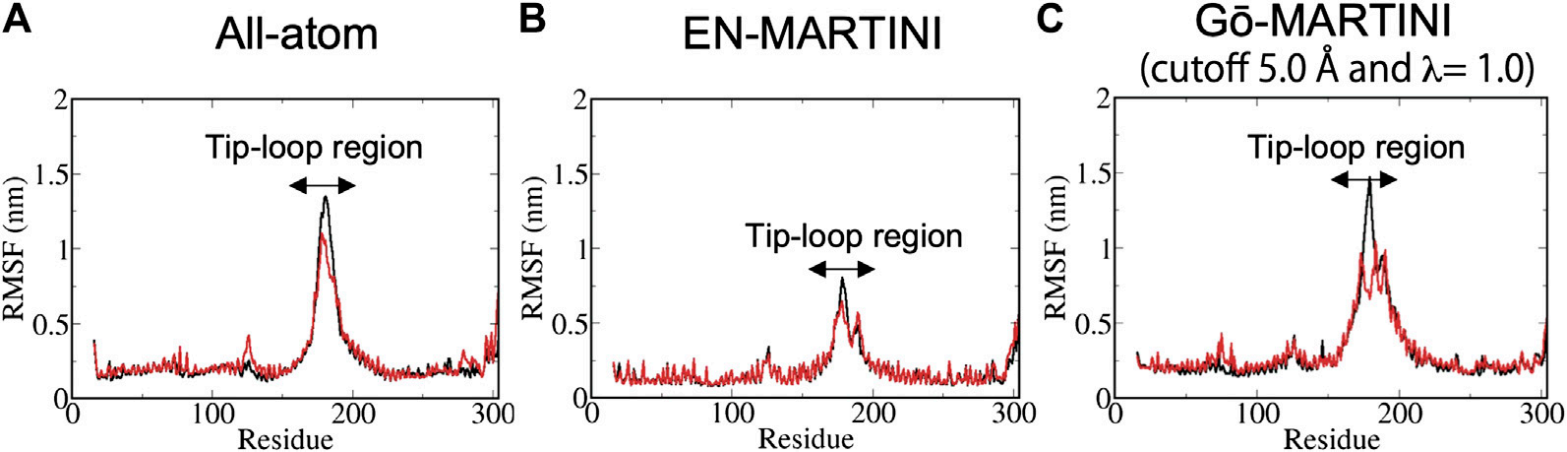
The RMSF results from **(A)** AA **(B)** EN-MARTINI **(C)** Gō-MARTINI (cutoff 5.0 Å and λ = 1.0) simulations are shown. For the RMSF calculation, the last half of the trajectories was used. Black and red lines represent chain A and B, respectively. Arrows indicates the tip-loop region in chain A and B.

The structural flexibility of a single Pacsin1 dimer on the lipid membrane was investigated through the AA and CG MD simulations (Figure 1). We carried out ∼500 ns AA simulation and ∼1000 ns CG simulations with the conventional EN-MARTINI and Gō-MARTINI (Figures 1A,B). First, we calculated the root mean squared fluctuation (RMSF) of the Pacsin1 dimer and compared it between the AA and CG simulations (see Figure 2). In this analysis, only the coordinates for backbone atoms of Pacsin1 were used. The RMSF represents the extent of amino acid residue fluctuation around their average positions. A comparison among simulations suggested that the fluctuation in the tip-loop region from the EN-MARTINI simulation is significantly underestimated compared to the AA simulation result (Figure 2). The underestimation of the RMSF in the tip-loop region is due to a limitation of the elastic network potential. To address this issue, we employed the Gō-MARTINI model, which can describe large-scale unfolding motions. We introduced a simple cutoff scheme to define the native contacts in the Gō model (see Methods). After exploring the Gō-MARTINI parameters, we found that the RMSF from the Gō-MARTINI simulation with the native contact cutoff 5.0 Å and interaction strength of the LJ potential λ = 1.0 is well fitted with the AA simulation result, including the tip-loop region residues (Figure 2). The RMSFs from the Gō-MARTINI simulations with the native contact cutoff values 4.5 and 5.5 Å or λ = 1.5 are slightly suppressed (see Supplementary Figures S2,3). In addition, the principal component analysis, Pacsin1 binding and assembly on the membrane support that the native contact cutoff 5.0 Å and λ = 1.0 is a best set of parameters, as we see below. These results indicate that choice of the force field parameters influence structural dynamics of the protein.

**FIGURE 3:**
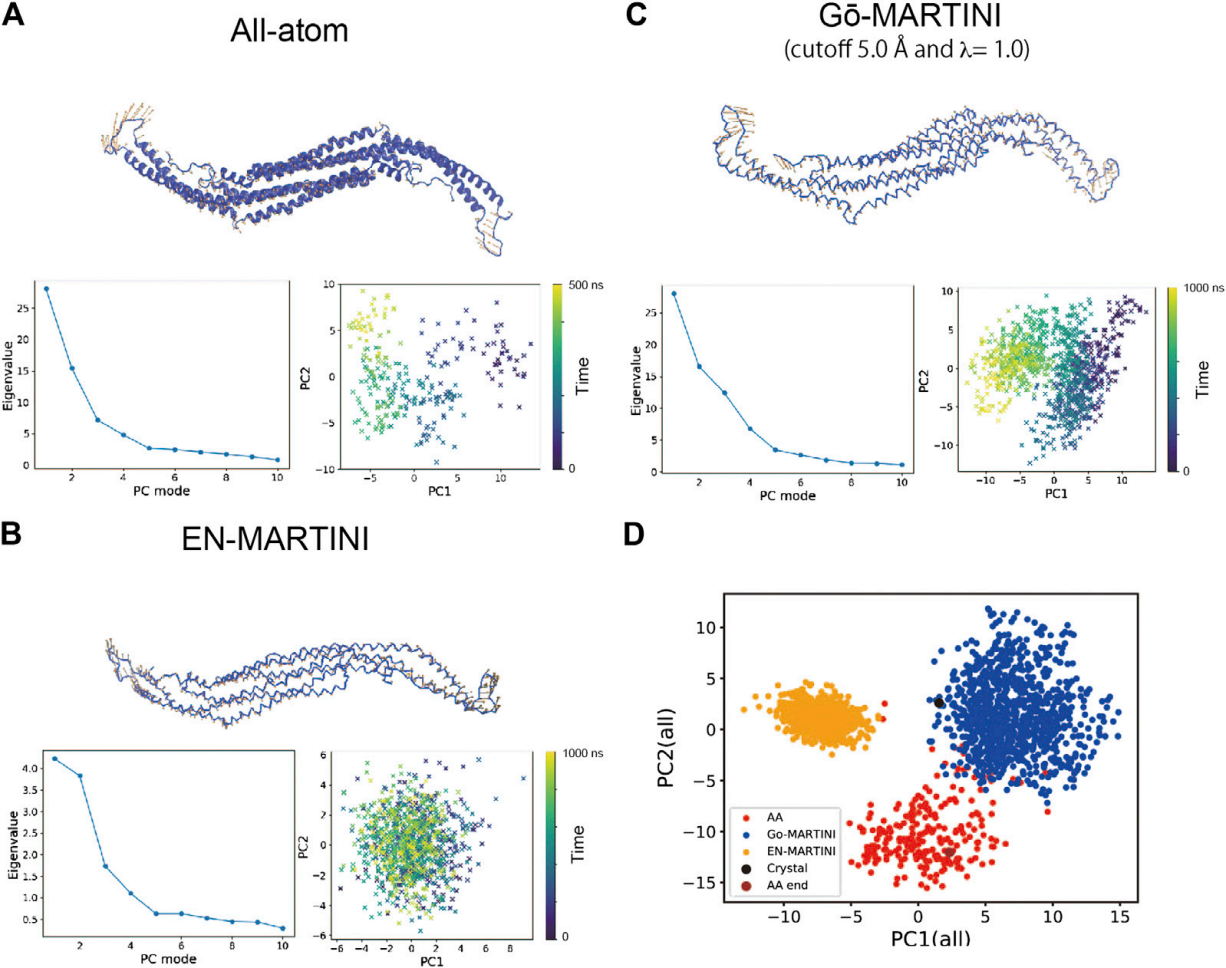
Principal component analysis (PCA) of the Pacsin1 structural fluctuations. For **(A)** All-atom **(B)** EN-MARTINI **(C)** Gō-MARTINI (cutoff 5.0 Å and λ = 1.0) results, the first principal component (PC1) eigenvector on the Pacsin1 structure, the eigenvalue profile along the principal component modes, and mapping of the Pacsin1 conformations on the PC1-PC2 surface are shown. Colorbars in the PC1-PC2 mapping represent the time progress in nanoseconds. In **(D)**, mapping of the Pacsin1 conformations on the PC1-PC2 surface obtained from PCA using all three simulations is shown.

Second, we performed the principal component analysis (PCA). The PCA identifies the axes of maximal variance of global structural fluctuations. The PCA was performed for trajectories from the AA and CG MARTINI MD simulations. In our analysis, we consider only Cα atoms of the protein. Figure 3 shows a visualization of the structural fluctuations from the first principal component mode and the eigenvalues along the principal component modes. The AA MD simulation shows that the tip-loop regions of the protein have high magnitude of fluctuations, which can be seen in the PC1 eigenvector. The PCA result from the Gō-MARTINI simulation with cutoff 5.0 Å, λ = 1.0 is in good agreement with the AA result, regarding not only the PC1 vector but also the eigenvalue profile along the PC modes. In contrast, the EN-MARTINI result shows an underestimated fluctuation, which is evident from the PC1 eigenvector and the eigenvalue profile. In order to compare the PCA results of the Gō-MARTINI and EN-MARTINI simulations to the reference AA result, we calculated RMSIP (see MATERIALS and METHODS) between the Gō-MARTINI and AA results, as well as between the EN-MARTINI and AA results. The RMSIP quantifies how much two simulation trajectories are overlapped in a subspace described by the first 10 eigenvectors. It was found that the RMSIP (Gō-MARTINI, AA) of 0.691 is higher than the RMSIP (EN-MARTINI, AA) of 0.652, indicating that the overlap between Gō-MARTINI and AA is better than that of EN-MARTINI and AA. We note that the time scale of 500 ns for the AA simulation might not be enough to fully cover slow conformational dynamics of the tip loops that contribute significantly to the global conformational changes. We also performed a single PCA using all three trajectories: AA, EN-MARTINI, and Gō-MARTINI (cutoff 5.0 Å, λ = 1.0), and projected each trajectory onto the common PC1 and PC2 (Figure 3D). The plot shows that conformations sampled in the AA and Gō-MARTINI overlap at the edges to some extent, while the EN-MARTINI samples an isolated, restricted region. The common PC1 and PC2 involve motions of the flexible tip loops (Supplementary Figure S4), which are expected to be slow and might not be fully covered by the 500 ns AA simulation. As the native contact cutoff of the Gō-MARTINI increases, magnitude of fluctuations decreases as seen from smaller eigenvalues (Supplementary Figure S5). Thus, the Gō-MARTINI with the native contact cutoff 5.0 Å and λ = 1.0 reproduces both local and global structural fluctuations of Pacsin1.

We also analyzed the interface structure between two chains of Pacsin1 during the simulations, because the native contacts (elastic bonds) were not considered for the interface in the current Gō-MARTINI (EN-MARTINI) simulations. We calculated the fraction of the virtual native contacts at the interface present during the simulations (Q_AB_, see Materials and Methods). The time courses of Q_AB_ for the Gō-MARTINI and EN-MARTINI simulations as well as the AA simulation are shown in Supplementary Figure S6. For the Gō-MARTINI models, the average value of Q_AB_ from the last half of the trajectory is 0.83 or higher, which indicates that the interface structure is basically maintained only with the MARTINI force field at the interface. For the EN-MARTINI model, we observed a similar average value of 0.86. These values are lower than the average value of 0.94 observed in the AA simulation. Note that the time scale of the AA simulation is much shorter than the Gō-MARTINI or EN-MARTINI simulations. This is ∼ 8 times shorter if we consider that MARTINI dynamics is faster than AA dynamics with the speed-up factor of ∼ 4 (Marrink et al., 2004). Using this factor and comparing all simulations in the same time scale, we can report a higher Q_AB_ value above 0.9 during the first 200 ns of the Gō-MARTINI (cutoff 5.0 Å and λ = 1.0) simulation, which would match the AA result.

### Pacsin1 Binding on the Lipid Membrane

**FIGURE 4:**
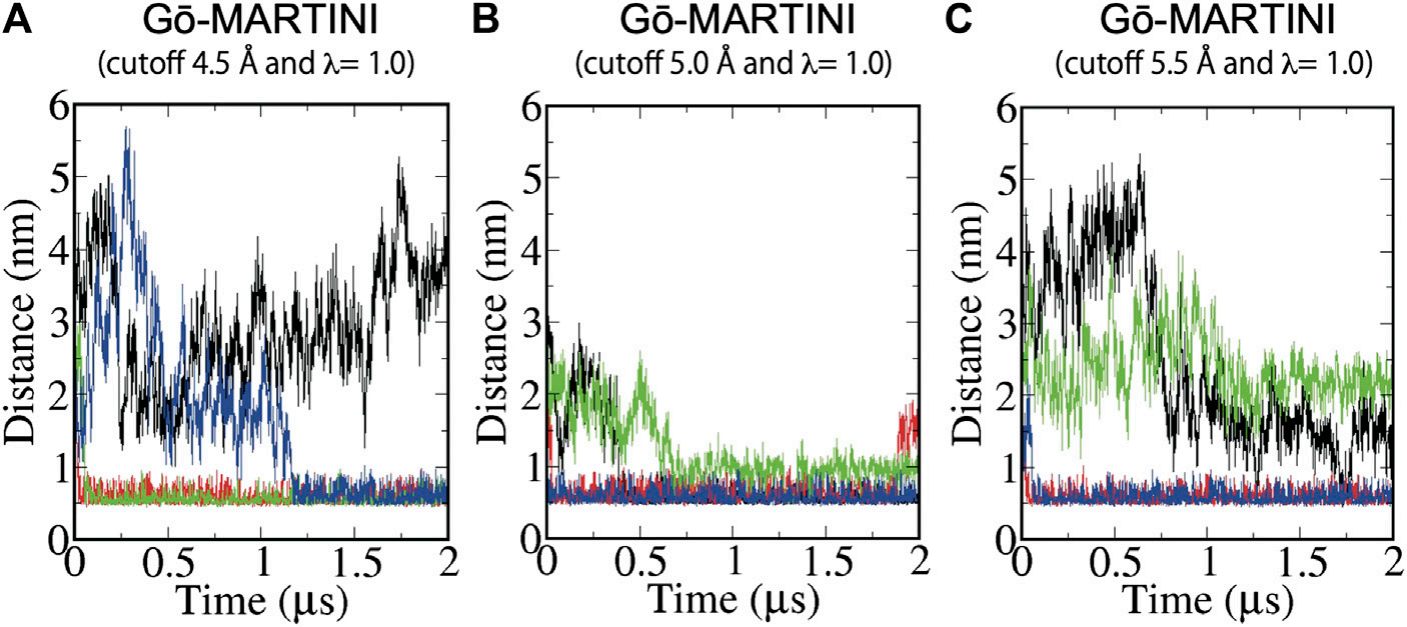
Binding of Pacsin1 to the membrane with the wedge loops inserted into the membrane. Distance between positively charged K123 of the wedge loop and negatively charged phosphate of the lipid head group during MD simulations of **(A)** Gō-MARTINI (cutoff 4.5 Å and λ = 1.0), **(B)** Gō-MARTINI (cutoff 5.0 Å and λ = 1.0), and **(C)** Gō-MARTINI (cutoff 5.5 Å and λ = 1.0) are shown. Black, red, green and blue lines represent the wedge loop 1, 2 of the first Pacsin1 and that of the second Pacsin1, respectively.

The structure of the F-BAR domain of Pacsin1 revealed distinctive wedge loops that are involved in the membrane binding and insertion (Wang et al., 2009). The wedge loop is a signature of Pacsin proteins and possibly affects their assembly (Bai et al., 2012). In our analysis, we found that positively charged residue Lys (K123) of the wedge loop interacts with negatively charged phosphate of the lipid head group during MD simulations. Thus, we calculated a minimum distance between K123 and the lipid phosphate as a measure of Pacsin1 binding. For the Gō-MARTINI (cutoff 4.5 Å, λ = 1.0), after a few nanoseconds, two wedge loops from different Pacsin1 dimers are inserted in the membrane (distance ∼0.5 nm) throughout the simulations. One of the remaining wedge loops is inserted in the membrane after 1.2 μs. The last one is not inserted in the membrane during the simulations. For the optimized Gō-MARTINI (cutoff 5.0 Å, λ = 1.0), we observed a clear interaction between the wedge loop and the membrane (Figure 4B). That is, the distance between the wedge loop (residue K123) and the membrane stayed close for all wedge-loops. In contrast, for the other Gō-MARTINI (cutoff 5.5 Å, λ = 1.0), two wedge loops from different Pacsin1 dimers are inserted in the membrane from the early stage of MD simulations (Figure 4C). But other two wedge loops are not inserted into the membrane and the distances stay larger than 1 nm.

### Pacsin1 Assembly Process on the Lipid Membrane

**FIGURE 5:**
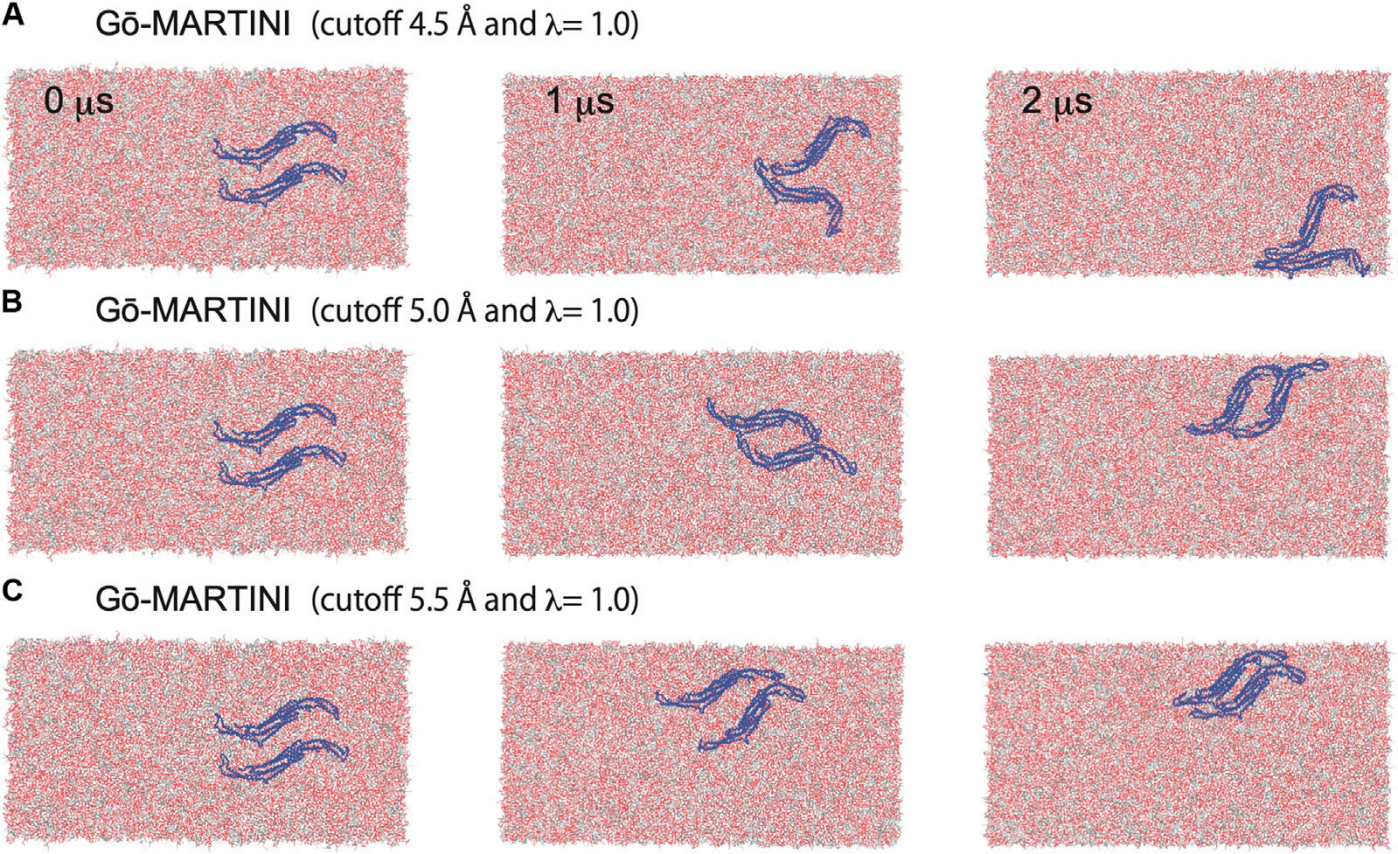
Pacsin1 assemblies on the lipid membrane simulated with the Gō-MARTINI (cutoff 4.5 Å and λ = 1.0), (cutoff 5.0 Å and λ = 1.0) and (cutoff 5.5 Å and λ = 1.0) are shown in **(A)**, **(B)** and **(C)**, respectively.

Assembly of Pacsin1 on the lipid membrane is one of the key features involved in the membrane remodeling. We carried out the Gō-MARTINI simulations with two Pacsin1 dimers on a flat tensionless membrane. During the 2 μs long simulation, stable Pacsin1-Pacsin1 lateral interaction was observed for the optimized Gō-MARTINI (cutoff 5.0 Å, λ = 1.0), while improper interactions were observed for the other Gō-MARTINIs (Figure 5). The lateral interaction observed in the optimized Gō-MARTINI was formed within a few nanoseconds and maintained throughout the simulation (Figure 5B). The similar lateral interaction was observed in the crystal structure of Pacsin1 (PDB entry, 3HAI) (Wang et al., 2009). Our previous study also revealed the similar lateral interaction of Pacsin1-Pacsin1 with the EN-MARTINI simulations (Mahmood et al., 2019). The inter-protein interaction is due to the physico-chemical interactions of the MARTINI force field. Thus, our results confirm that protein-protein interactions are well described by MARTINI (Baaden and Marrink, 2013). More importantly, it was also demonstrated that the observed inter-protein interactions, with the same MARTINI force field describing them, are strictly dependent on the definition of the intra-protein potentials.

## Discussion

In this study, we have adapted the Gō-MARTINI model to describe structural dynamics and assembly of the F-BAR protein Pacsin1. We introduced a simple cutoff scheme for definition of the native contacts instead of the OV + rCSU approach used in the original Gō-MARTINI (Poma et al., 2017). The cutoff scheme is more flexible and allows us to explore parameters such as the cutoff distance of the native contacts. The optimized Gō-MARTINI simulations reproduce global and local structural fluctuations from the AA simulation. The transferability of the current scheme including the cutoff distance of the native contacts should be tested with other systems to build a universal model. It was also shown that Pacsin1 binding and assembly on the membrane were reproduced properly by the optimized Gō-MARTINI. These results suggest that protein-lipid and protein-protein interactions are well described by the physico-chemical MARTINI force field, once proper intra-protein structures are prepared. The earlier success of the EN-MARTINI model for protein-protein interactions supports this notion (Baaden and Marrink, 2013). However, large conformational changes of intra-protein structures are beyond the scope of the EN-MARTINI model. Our results show that EN-MARTINI can be replaced by Gō-MARTINI, and the Gō-MARTINI model performs better than the EN-MARTINI model in terms of intra-protein structural fluctuations. The Gō-MARTINI model also offers advantages over the commonly used bond-angle restrained MARTINI, which maintains the local secondary structures. The bond-angle restrained MARTINI has been used for rather small or flexible proteins, such as helical peptides (Monticelli et al., 2008) or α-synuclein (Braun et al., 2012). However, this model is not applicable to proteins that have specific native structures more complicated than a single helix. Thus, the Gō-MARTINI model has advantages in simulating conformational dynamics of proteins with the specific native structures.

We note that the protein model in Gō-MARTINI is not a pure “Gō model”, because it has non-native (that is, physico-chemical) interactions from the MARTINI force field. Previous works on protein-protein interactions showed that the MARTINI force field tends to overestimate protein-protein interactions, and thus, down-scaling of the interactions is necessary to reproduce experimental results (Stark et al., 2013; Javanainen et al., 2017; Benayad et al., 2020). By optimizing both the structure-based Gō interactions (Li et al., 2011, 2012) and physico-chemical MARTINI interactions (Alessandri et al., 2019), we have a unique opportunity to properly describe intra and inter protein structural stability and dynamics with the Gō-MARTINI model. Possible improvements of the structure-based Gō interactions include refinement of contact energy in a residue-pair specific manner. The previously developed methods such as atomic-interaction-based coarse-grained (AICG) model (Li et al., 2011) or Miyazawa-Jernigan statistical contact energy (Karanicolas and Brooks, 2002) can be used. The definition of the native contacts itself can be improved by symmetrizing between homodimers or analyzing the contacts in the all-atom simulations instead of the static experimental structure. The dynamic contact analysis of the all-atom simulations discerns between stable and transient contacts (Moreira et al., 2020), where the latter can be excluded from the native contacts. Another improvement would be an extension of the current single-basin Gō model to a multiple-basin Gō model to explore conformational changes between different stable conformations such as ligand-free and bound conformations. The previous methods such as the multiple-basin energy landscape model (Okazaki et al., 2006) or the double-well ultra-coarse-grained model (Zhang et al., 2020) can be introduced.

To describe realistic dynamics of membrane remodeling in the Gō-MARTINI framework, we might need a further reduction of the dimension of the model. The simulation system can become very large when whole membrane remodeling processes are considered with large membranes and many proteins involved. One possible way to reduce the dimension in Gō-MARTINI is to replace normal MARTINI with Dry MARTINI, an implicit solvent version of MARTINI (Arnarez et al., 2015). This is a highly effective approach because solvent beads dominate the total number of beads as the simulation system becomes large. Although there are some modifications of the force field parameters in Dry MARTINI, our Gō-MARTINI framework is expected to apply with possible minor changes. In addition, an extreme reduction to a continuum membrane model and backmapping to the MARTINI model has been explored recently to simulate membrane transformation of an entire mitochondrion (Pezeshkian et al., 2020). When this type of multiscale approach is combined with an accurate description of membrane-protein system at the molecular level by Gō-MARTINI, it will be a powerful tool to simulate large systems of membrane remodeling processes.

## Data Availability

The raw data supporting the conclusions of this article will be made available by the authors, without undue reservation.
