# Supplementary material for: Optimizing Gō-MARTINI Coarse-Grained Model for F-BAR Protein on Lipid Membrane
Source: Front Mol Biosci. 2021 Feb 22;8:619381. doi: 10.3389/fmolb.2021.619381 (PMC7937874; doi:10.3389/fmolb.2021.619381)
Supplement: Supplementary file 1 [file datasheet1.pdf]

Supplementary Material

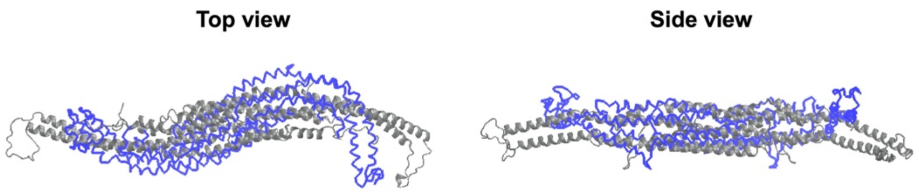

Supplementary Figure 1. Final snapshots of Pacsin1 from the Gō-MARTINI (OV+rCSU) simulation are shown from the top and side views in blue, while those of Pacsin1 from the all-atom simulation are also shown in gray.

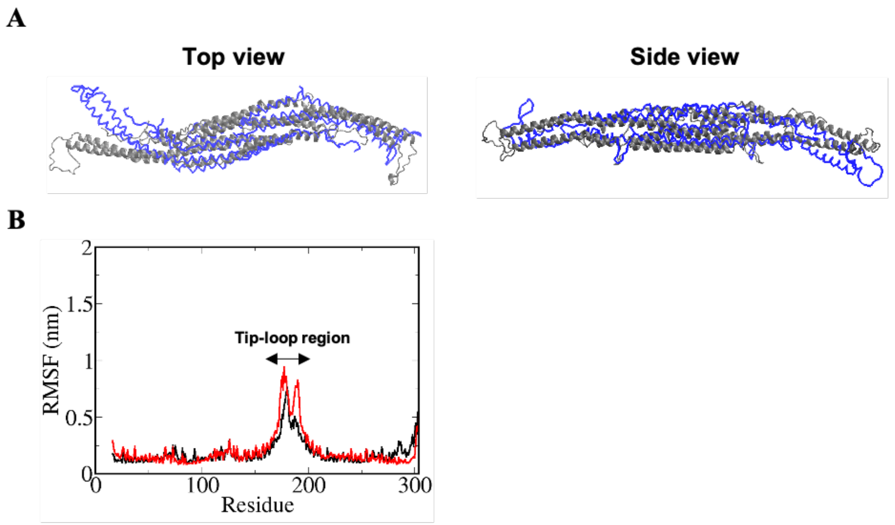

Supplementary Figure 2. **(A)** Final snapshots of Pacsin1 from the Gō-MARTINI (cutoff 5.0 Å and $\lambda = 1.5$) simulation are shown from the top and side views, while those of Pacsin1 from the all-atom simulation are also shown in gray. **(B)** The RMSF result from the Gō-MARTINI (cutoff 5.0 Å and $\lambda = 1.5$) simulation is shown. For the RMSF calculation, the last half of the trajectories was used. Black and red lines represent chain A and B, respectively. Arrows indicates the tip-loop region in chain A and B.

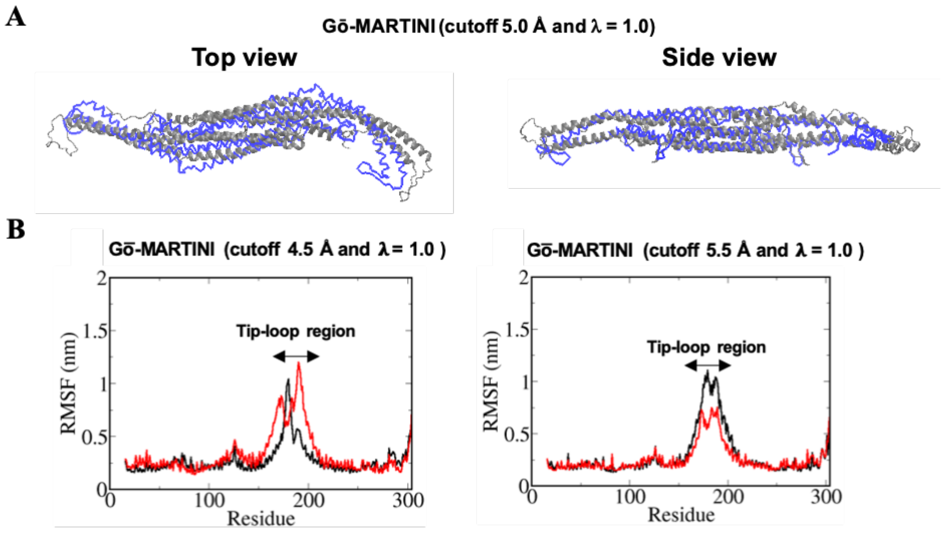

Supplementary Figure 3. **(A)** Final snapshots of Pacsin1 from the Gō-MARTINI (cutoff 5.0 Å and λ = 1.0) simulation are shown from the top and side views, while those of Pacsin1 from the all-atom simulation are also shown in gray. **(B)** The RMSF results from Gō-MARTINI (cutoff 4.5 Å and λ = 1.0) and Gō-MARTINI (cutoff 5.5 Å and λ = 1.0) simulations are shown. For the RMSF calculation, the last half of the trajectories was used. Black and red lines represent chain A and B, respectively. Arrows indicates the tip-loop region in chain A and B.

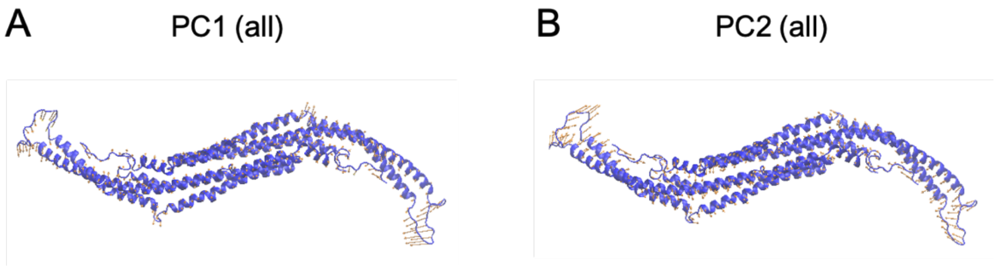

Supplementary Figure 4. **(A)** The common PC1 eigenvector is shown on Pacsin1 structure. **(B)** The common PC2 eigenvector is shown on Pacsin1 structure.

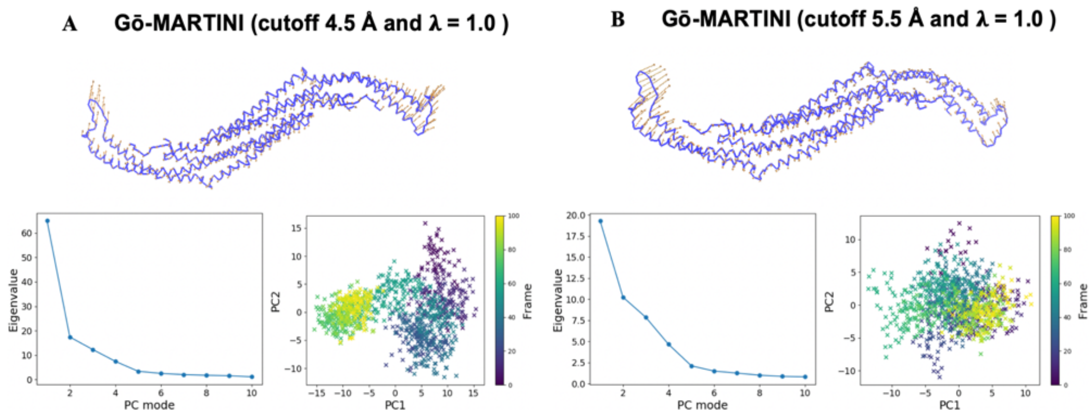

Supplementary Figure 5. Principle component analysis of the Pacsin1 structural fluctuations. For **(A)** Gō-MARTINI (cutoff 4.5 Å and λ = 1.0) and **(B)** Gō-MARTINI (cutoff 5.5 Å and λ = 1.0) simulations, the first principal component (PC1) eigenvector on the Pacsin1 structure, the eigenvalue profile along the principal component modes, and mapping of the Pacsin1 conformations on the PC1-PC2 surface are shown.

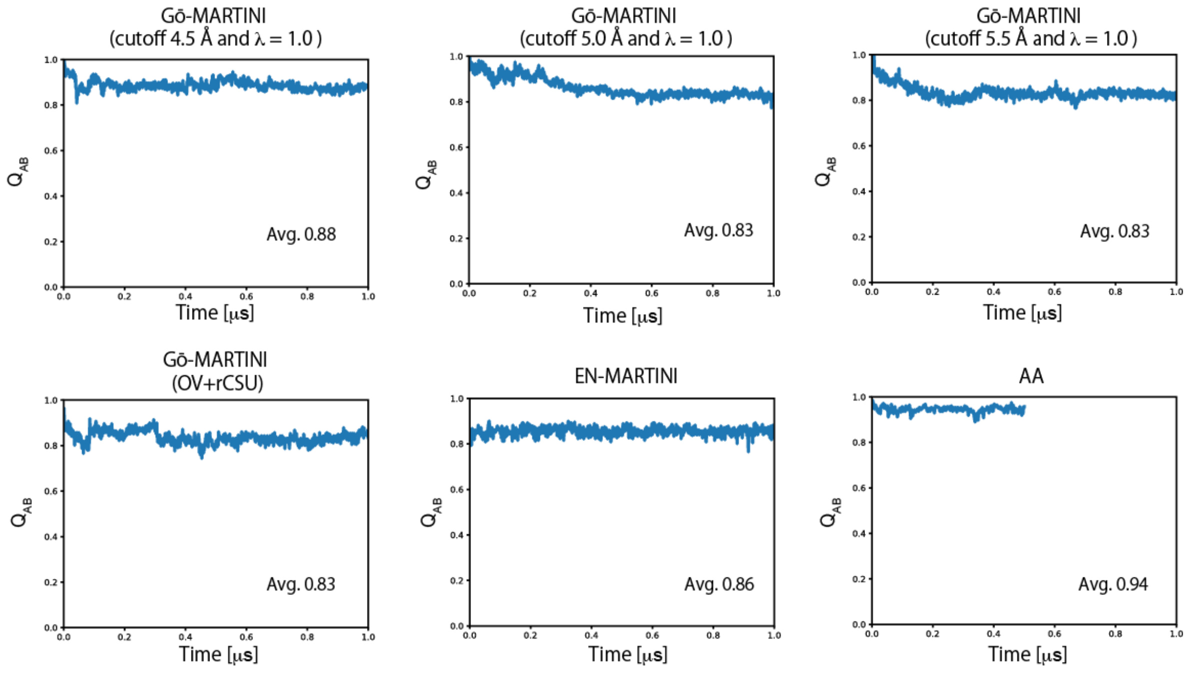

Supplementary Figure 6. The fraction of the virtual native contacts at the interface between chain A and chain B present during simulations of the tested Gō-MARTINI and EN-MARTINI models as well as the AA model. The average value of Q_{AB} from the last half of the simulations is shown in the inset.

Model	EN-MARTINI	Gō-MARTINI (OV+rCSU)	Gō-MARTINI (cutoff 4.5 Å)	Gō-MARTINI (cutoff 5.0 Å)	Gō-MARTINI (cutoff 5.5 Å)
Number of native contacts (chain A)	2668	469	757 (96.8% overlap)	856 (98.5% overlap)	1021 (99.4% overlap)
Number of native contacts (chain B)	2646	461	746 (97.4% overlap)	843 (97.7% overlap)	1009 (98.2% overlap)

Supplementary Table 1: Number of native contacts in different Gō-MARTINI and EN-MARTINI models. Numbers in parenthesis indicate percentage of the overlapped native contacts from the Gō-MARTINI (OV+rCSU).
